# Supplementary material for: Modeling glioblastoma heterogeneity as a dynamic network of cell states
Source: Mol Syst Biol. 2021 Sep 16;17(9):e10105. doi: 10.15252/msb.202010105 (PMC8444284; doi:10.15252/msb.202010105)
Supplement: Supplementary file 6 — Source Data for Figure 5 [file MSB-17-e10105-s004.zip › Figure5A_sourcedata/GSEA_3017/nosigdb_stateB.GseaPreranked.1622795021725/LARSSON_STATE_4.html]

Details for gene set LARSSON\_STATE\_4[GSEA]

|  || Dataset | state43017 |
| Phenotype | NoPhenotypeAvailable |
| Upregulated in class | na\_pos |
| GeneSet | LARSSON\_STATE\_4 |
| Enrichment Score (ES) | 0.6641324 |
| Normalized Enrichment Score (NES) | 3.736739 |
| Nominal p-value | 0.0 |
| FDR q-value | 0.0 |
| FWER p-Value | 0.0 |
Table: GSEA Results Summary

  

Fig 1: Enrichment plot: LARSSON\_STATE\_4      
 Profile of the Running ES Score & Positions of GeneSet Members on the Rank Ordered List

  

| PROBE | GENE SYMBOL | GENE\_TITLE | RANK IN GENE LIST | RANK METRIC SCORE | RUNNING ES | CORE ENRICHMENT || 1 | HIST1H4C |  |  | 0 | 1.373 | 0.0302 | Yes |
| 2 | HIST1H1B |  |  | 1 | 1.272 | 0.0582 | Yes |
| 3 | HIST1H1D |  |  | 2 | 1.180 | 0.0841 | Yes |
| 4 | TOP2A |  |  | 3 | 1.079 | 0.1078 | Yes |
| 5 | HIST1H1C |  |  | 4 | 1.041 | 0.1307 | Yes |
| 6 | HIST1H1E |  |  | 5 | 0.983 | 0.1524 | Yes |
| 7 | RRM2 |  |  | 7 | 0.875 | 0.1701 | Yes |
| 8 | UBE2C |  |  | 11 | 0.802 | 0.1834 | Yes |
| 9 | TPX2 |  |  | 12 | 0.801 | 0.2010 | Yes |
| 10 | NUSAP1 |  |  | 13 | 0.801 | 0.2186 | Yes |
| 11 | HMGB2 |  |  | 14 | 0.786 | 0.2359 | Yes |
| 12 | FBXO5 |  |  | 15 | 0.785 | 0.2531 | Yes |
| 13 | NCAPG |  |  | 16 | 0.778 | 0.2702 | Yes |
| 14 | ESCO2 |  |  | 20 | 0.742 | 0.2822 | Yes |
| 15 | GTSE1 |  |  | 22 | 0.732 | 0.2968 | Yes |
| 16 | ATAD2 |  |  | 28 | 0.685 | 0.3045 | Yes |
| 17 | KIF15 |  |  | 29 | 0.684 | 0.3195 | Yes |
| 18 | PCLAF |  |  | 31 | 0.681 | 0.3330 | Yes |
| 19 | SGO1 |  |  | 32 | 0.677 | 0.3479 | Yes |
| 20 | KPNA2 |  |  | 33 | 0.676 | 0.3628 | Yes |
| 21 | CENPF |  |  | 34 | 0.670 | 0.3775 | Yes |
| 22 | SGO2 |  |  | 36 | 0.664 | 0.3907 | Yes |
| 23 | KIF4A |  |  | 37 | 0.662 | 0.4052 | Yes |
| 24 | RAD51AP1 |  |  | 40 | 0.657 | 0.4167 | Yes |
| 25 | SMC4 |  |  | 41 | 0.655 | 0.4311 | Yes |
| 26 | DHFR |  |  | 49 | 0.638 | 0.4349 | Yes |
| 27 | FANCI |  |  | 56 | 0.615 | 0.4396 | Yes |
| 28 | MYBL2 |  |  | 58 | 0.612 | 0.4516 | Yes |
| 29 | CENPU |  |  | 60 | 0.605 | 0.4634 | Yes |
| 30 | CENPK |  |  | 62 | 0.603 | 0.4752 | Yes |
| 31 | TYMS |  |  | 63 | 0.601 | 0.4884 | Yes |
| 32 | SMC2 |  |  | 68 | 0.586 | 0.4954 | Yes |
| 33 | CDK1 |  |  | 69 | 0.585 | 0.5083 | Yes |
| 34 | H2AFX |  |  | 71 | 0.576 | 0.5195 | Yes |
| 35 | CKS1B |  |  | 72 | 0.575 | 0.5321 | Yes |
| 36 | MAD2L1 |  |  | 77 | 0.569 | 0.5388 | Yes |
| 37 | DBF4 |  |  | 79 | 0.567 | 0.5498 | Yes |
| 38 | CKS2 |  |  | 83 | 0.558 | 0.5576 | Yes |
| 39 | UBE2S |  |  | 87 | 0.555 | 0.5654 | Yes |
| 40 | CENPM |  |  | 92 | 0.544 | 0.5715 | Yes |
| 41 | CCNB1 |  |  | 93 | 0.544 | 0.5835 | Yes |
| 42 | PBK |  |  | 95 | 0.540 | 0.5939 | Yes |
| 43 | RRM1 |  |  | 103 | 0.520 | 0.5950 | Yes |
| 44 | CDKN3 |  |  | 108 | 0.513 | 0.6004 | Yes |
| 45 | UBE2T |  |  | 110 | 0.512 | 0.6102 | Yes |
| 46 | BIRC5 |  |  | 116 | 0.504 | 0.6140 | Yes |
| 47 | CLSPN |  |  | 117 | 0.503 | 0.6250 | Yes |
| 48 | DTYMK |  |  | 118 | 0.503 | 0.6361 | Yes |
| 49 | ASF1B |  |  | 120 | 0.501 | 0.6456 | Yes |
| 50 | DUT |  |  | 124 | 0.494 | 0.6521 | Yes |
| 51 | TMPO |  |  | 127 | 0.492 | 0.6600 | Yes |
| 52 | GMNN |  |  | 135 | 0.484 | 0.6603 | Yes |
| 53 | ORC6 |  |  | 150 | 0.461 | 0.6499 | Yes |
| 54 | CDC45 |  |  | 154 | 0.456 | 0.6555 | Yes |
| 55 | TK1 |  |  | 167 | 0.444 | 0.6477 | Yes |
| 56 | PIMREG |  |  | 169 | 0.443 | 0.6559 | Yes |
| 57 | BRCA1 |  |  | 171 | 0.440 | 0.6641 | Yes |
| 58 | MKI67 |  |  | 179 | 0.436 | 0.6634 | No |
| 59 | MND1 |  |  | 193 | 0.424 | 0.6537 | No |
| 60 | MCM4 |  |  | 199 | 0.418 | 0.6555 | No |
| 61 | ZWINT |  |  | 212 | 0.407 | 0.6468 | No |
| 62 | H2AFZ |  |  | 216 | 0.405 | 0.6513 | No |
| 63 | EZH2 |  |  | 236 | 0.388 | 0.6320 | No |
| 64 | PCNA |  |  | 242 | 0.386 | 0.6331 | No |
| 65 | HMGN2 |  |  | 243 | 0.386 | 0.6416 | No |
| 66 | TUBB |  |  | 263 | 0.368 | 0.6218 | No |
| 67 | CHAF1A |  |  | 276 | 0.359 | 0.6121 | No |
| 68 | CENPN |  |  | 309 | 0.343 | 0.5726 | No |
| 69 | DEK |  |  | 316 | 0.341 | 0.5713 | No |
| 70 | PHF19 |  |  | 319 | 0.339 | 0.5758 | No |
| 71 | RFC2 |  |  | 331 | 0.333 | 0.5670 | No |
| 72 | LMNB1 |  |  | 363 | 0.321 | 0.5285 | No |
| 73 | PTTG1 |  |  | 413 | 0.302 | 0.4632 | No |
| 74 | NUCKS1 |  |  | 483 | 0.285 | 0.3682 | No |
| 75 | CENPH |  |  | 501 | 0.281 | 0.3494 | No |
| 76 | LSM3 |  |  | 522 | 0.277 | 0.3261 | No |
| 77 | ALYREF |  |  | 546 | 0.273 | 0.2983 | No |
| 78 | RPA3 |  |  | 584 | 0.267 | 0.2499 | No |
| 79 | UHRF1 |  |  | 652 | 0.255 | 0.1571 | No |
Table: GSEA details [plain text format]

  

Fig 2: LARSSON\_STATE\_4: Random ES distribution      
 Gene set null distribution of ES for **LARSSON\_STATE\_4**
